# Supplementary material for: Enterocytes, fibroblasts and myeloid cells synergize in anti-bacterial and anti-viral pathways with IL22 as the central cytokine
Source: Commun Biol. 2021 May 27;4:631. doi: 10.1038/s42003-021-02176-0 (PMC8160143; doi:10.1038/s42003-021-02176-0)
Supplement: Supplementary file 2 — Supplementary Information [file 42003_2021_2176_MOESM2_ESM.pdf]

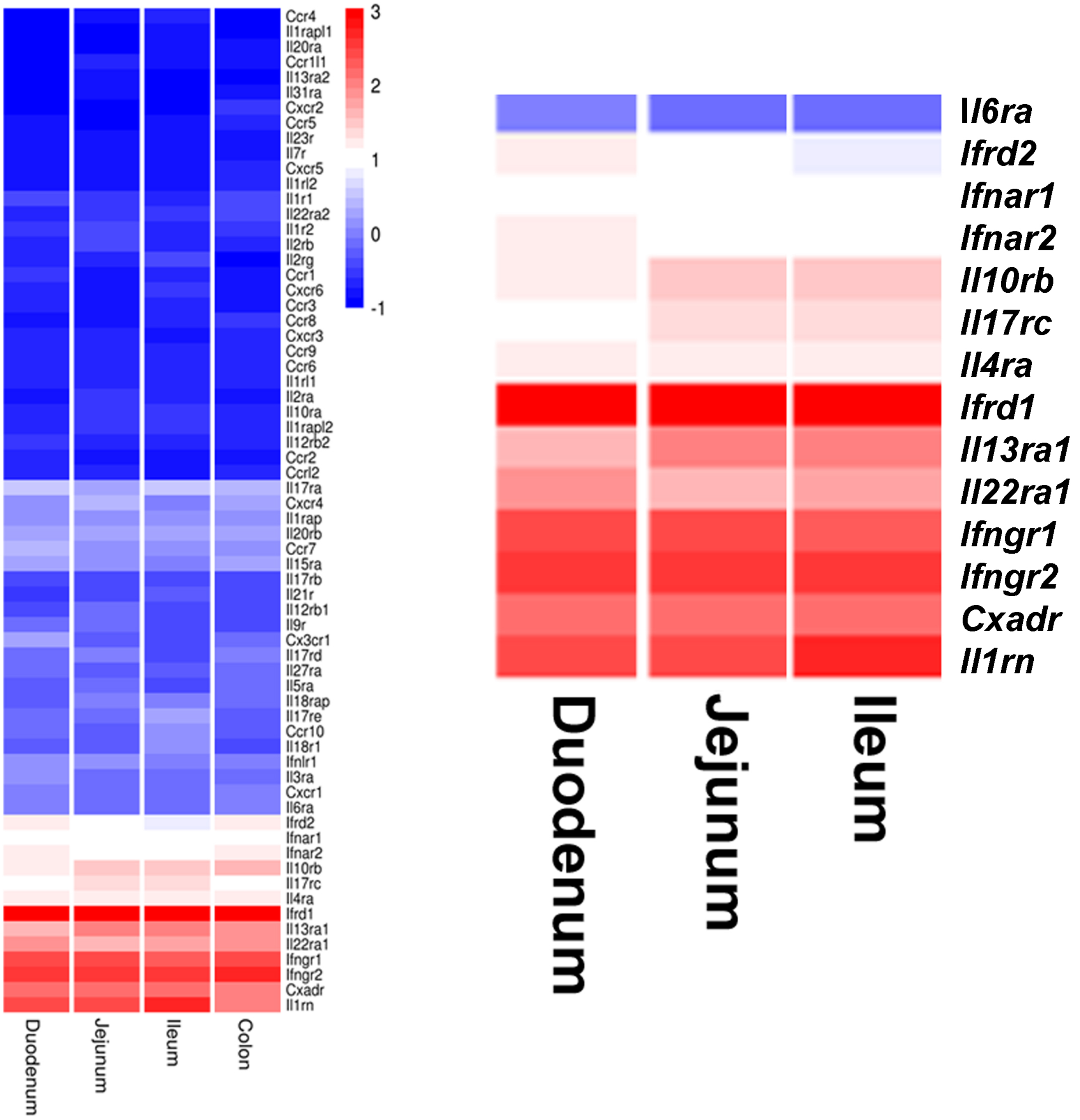

Figure S1 *Organoid array cytokine receptors.*

Cytokine receptor mRNA expression in duodenum, jejunum and ileum-derived organoids.

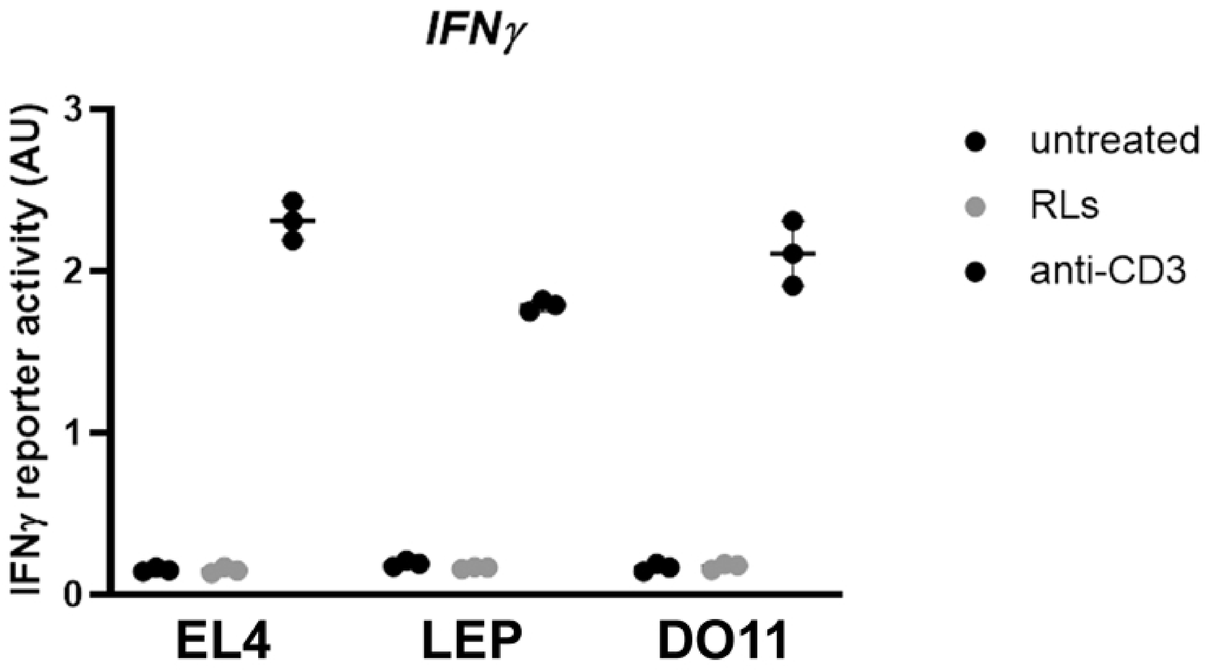

Figure S2 *IFNγ production by EL4, LEP and DO11 cells.*

Reporter activity assay for IFNγ on supernatants from EL4, LEP or DO11 T cells which were exposed to supernatants from LPS-stimulated RAW264.7 cells (RLs) for 48h. All experiments were performed in triplicate with a minimum of 3 independent experiments. Data are shown as mean ±SD. For statistical analyses, Log2 transformed data were used in Welch and Brown-Forsythe tests followed by Dunnett's T3 multiple comparisons test. * $P < 0.05$ was considered to indicate statistical significance.

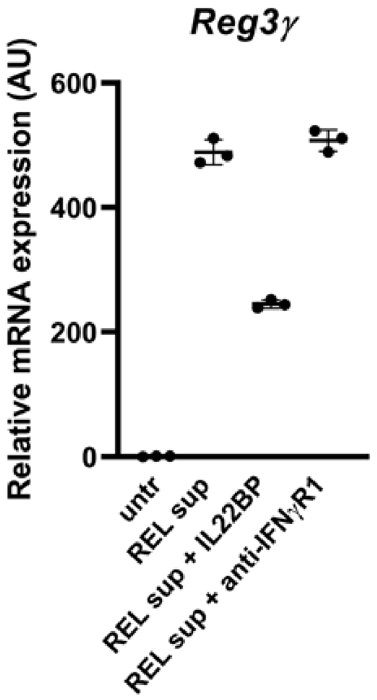

Figure S3 *IL22BP and anti-IFN-γR1 inhibit REL-induced Reg3γ expression in mouse Ileum-derived organoids.*

Reg3γ mRNA expression in ileum-derived organoids after exposure of EL4-derived supernatants with prior exposure to LPS-treated RAW264.7 cell supernatant (REL) in the presence or absence of recombinant mouse IL22BP (50 ng/ml) or blocking antibodies IFNγR1. All experiments were performed in triplicate with a minimum of 3 independent experiments. Data are shown as mean ±SD. For statistical analyses, Log2 transformed data were used in Welch and Brown-Forsythe tests followed by Dunnett's T3 multiple comparisons test. * $P < 0.05$ was considered to indicate statistical significance.

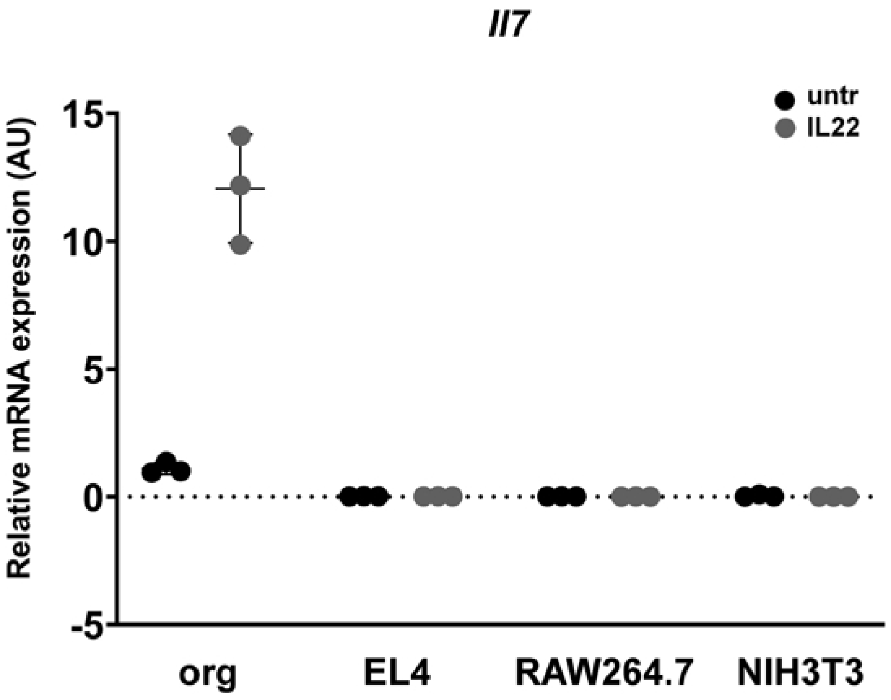

Figure S4 *Il7 mRNA expression in Ileum organoids, EL4, RAW264.7 and NIH3T3.*

Il7 mRNA expression in 3D mouse organoids, EL4, RAW264.7 and NIH3T3 after IL22 (5 ng/ml) exposure for 24h. All experiments were performed in triplicate with a minimum of 3 independent experiments. Data are shown as mean ±SD. For statistical analyses, Log2 transformed data were used in Welch and Brown-Forsythe tests followed by Dunnett's T3 multiple comparisons test. * $P < 0.05$ was considered to indicate statistical significance.

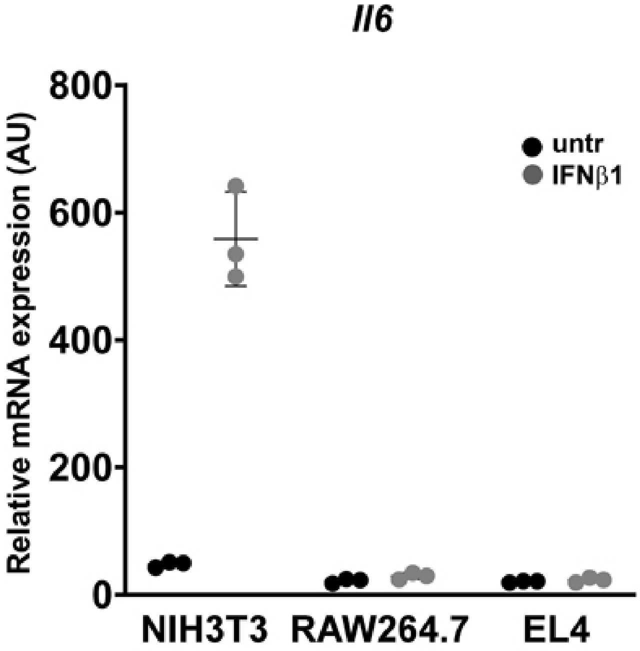

Figure S5 *Il6 mRNA expression in NIH3T3, RAW264.7 and EL4 cells.*

Il6 mRNA expression in NIH3T3, RAW264.7 and EL4 cells after IFNβ1 (20 ng/ml) exposure for 24h. All experiments were performed in triplicate with a minimum of 3 independent experiments. Data are shown as mean ±SD. For statistical analyses, Log2 transformed data were used in Welch and Brown-Forsythe tests followed by Dunnett's T3 multiple comparisons test. * $P < 0.05$ was considered to indicate statistical significance.

Gene	Function
Rsad2/Viperin	inhibitor viral RNA polymerase
Ifit 1-3	anti-alphavirus effectors
Pvr Cd155	activating cytotoxic lymphocytes via DNAM-1 receptor
Zap	anti-alphavirus effectors
Ace2	MERS entry
Tmprss2	SARS-Cov and SARS-Cov-2 entry
Dpp4	SARS-Cov and SARS-Cov-2 entry
Tnfrsf14	herpesvirus entry
Cd46	Measles virus entry
Slamf1/Cdw150	Measles virus entry
nectin-1/Pvrl1/Cd111	HveC (herpesvirus) entry
nectin-2/Pvrl2/Cd112	HveB (herpesvirus) entry
nectin-4/Pvrl4	MV entry
necl-5/Cd155/Pvr	Poliovirus entry
Xpr1	xenotropic and polytropic retrovirus receptor 1
Fv1	inhibit retroviral capsid
Trim5	inhibit retroviral capsid
Apobec1	destabilize retroviral genome
Apobec3	destabilize retroviral genome
Fut2	Norovirus entry (HBGA)
Cd300lf	Norovirus entry (HBGA)
Havcr1	hepatitis A virus cellular receptor 1
Havcr2	hepatitis A virus cellular receptor 2
Ivns1abp	influenza virus NS1A binding protein
Shfl	antiviral inhibitor of ribosomal frameshifting
Zc3hav1	zinc finger CCCH type, antiviral 1
Rig-I/Ddx58	MV recognition receptor
Ifih1/Mda-5	MV recognition receptor
Mx1/2	antiviral state against influenza virus infection
Oas	Ribonuclease
Gbp1	suppresses CSFV replication
Ifi6	Blocks Flavivirus Replication
Ifi27	Restricts West Nile Virus Infection
Isg20	degradation of viral RNA
Pkr/EIf2ak2	inhibits cellular mRNA translation
Ntcp	entry receptor for hepatitis B and D viruses
ScarB1	Dengue entry
Apoa1	Dengue entry

Table S1 *Overview of genes involved in viral entry and defense.*

	Forward	Reverse
Actb	TGGATGACGATATCGCTGCG	AGGGTCAGGATACCTCTCTT
Il22	AAGCTGCATGCTCACAGTGC	GGAGGTGGTACCTTTCCTGA
Il22	CGCTGCCCGTCAACACCCGG	CTGATCTTTAGCACTGACTCCTCG
Il22	CCAGCCTTGCAGATAACAAC	GGAAGGAGCAGTTCTTCGT
Ifnα	ACCTCAGGAACAAGAGAGCC	CTGCGGGAATCCAAAGTCCT
Ifnβ	TAAGCAGCTCCAGCTCCAAG	CCCTGTAGGTGAGGTTGATC
Ifnγ	GGATGCATTCATGAGTATTGC	CTTTTCCGCTTCCTGAGG
Il6	ACGATGATGCACTTGCAGA	GAGCATTGGAAATTGGGGTA
Il7	TCTGCTGCCTGTCACATCATC	GGACATTGAATTCTTCACTGATATTCA
Tnfα	GCCTCTTCTCATTCCTGCTTGT	TTGAGATCCATGCCGTTG
Saa1	GCTACTCACCAGCCTGGTCT	GGCCTCTCTTCCATCACTGA
Reg3β	GTAACAGTGGCCAATATGTATGGA	TACTCTAGGCCTTGAATTTGCAG
Reg3γ	AGAAGCATTTCTCAGGACACCT	CAGTTATAGAAGGTCAGAGGGTCAG
Hprt	AGTGTTGGATACAGGCCAGAC	CGTGATTCAAATCCCTGAAGT
Il1α	GCTTGACGTTGCTGATACTG	CAAGATGGCCAAAGTTCCTG

Table S2 *QPCR primers*
