# Supplementary material for: Enterocytes, fibroblasts and myeloid cells synergize in anti-bacterial and anti-viral pathways with IL22 as the central cytokine
Source: Commun Biol. 2021 May 27;4:631. doi: 10.1038/s42003-021-02176-0 (PMC8160143; doi:10.1038/s42003-021-02176-0)
Supplement: Supplementary file 3 — Description of Additional Supplementary Files [file 42003_2021_2176_MOESM3_ESM.pdf]

**Description of Additional Supplementary Files**

**File name:** Supplementary Data 1

**Description:** Expression analyses of *Reg3$\gamma$* mRNA in intestinal organoids after exposure to supernatants of LPS-stimulated immune cells.

**File name:** Supplementary Data 2

**Description:** T-cells can produce IL22 which subsequently can stimulate the expression of *Reg3$\gamma$* in small-intestinal organoids.

**File name:** Supplementary Data 3

**Description:** Similar to the T-cell line EL4, we also show that freshly isolated intestinal epithelial cells can produce IL22 when exposed to supernatants of LPS-activated dendritic cells.

**File name:** Supplementary Data 4

**Description:** IL22 secretion by EL4 T-cells can be induced by IL6 and strongly enhanced by either TNF$\alpha$ or IL7.

**File name:** Supplementary Data 5

**Description:** Combined cultures of macrophages and fibroblasts produce higher levels then single cultures. This enhancement is dependent on IL1$\alpha$/IL$\beta$ and TNF$\alpha$.

**File name:** Supplementary Data 6

**Description:** After exposure to LPS, intestinal organoids can secrete SAA3, which can trigger a cytokine cascade with IL1$\alpha$, IL1$\beta$ and IL6 in fibroblasts and macrophage and subsequently IL22 in EL4 T-cells.

**File name:** Supplementary Data 7

**Description:** Virus-induced IFN$\beta$1 in intestinal organoids can trigger a cytokine cascade, with IL7 being produced by enterocytes and IL6 by fibroblasts, resulting in IL22 secretion by EL4 T-cells. Furthermore, IL22 enhances the expression of anti-viral proteins and blocks the lateral spread of mouse coronavirus (MHV).
